# Dynamics of the association between circulating levels of miRNA and pancreatic cancer risk through the years prior to pancreatic cancer diagnosis

**DOI:** 10.1002/ijc.70452

**Published:** 2026-03-17

**Authors:** Hui Cai, Veronica Wendy Setiawan, Xingyi Guo, Jie Wu, Rachael Stolzenberg‐Solomon, Yu‐Tang Gao, Jordan Berlin, Fei Ye, Qiuyin Cai, Wei Zheng, Xiao‐Ou Shu

**Affiliations:** ^1^ Division of Epidemiology, Department of Medicine Vanderbilt University Medical Center Nashville Tennessee USA; ^2^ Department of Population and Public Health Sciences, Keck School of Medicine University of Southern California Los Angeles California USA; ^3^ Metabolic Epidemiology Branch, Division of Cancer Epidemiology and Genetics National Cancer Institute Rockville Maryland USA; ^4^ Department of Epidemiology, Shanghai Cancer Institute, Renji Hospital Shanghai Jiaotong University School of Medicine Shanghai China; ^5^ Division of Hematology and Oncology, Department of Medicine Vanderbilt University Medical Center Nashville Tennessee USA; ^6^ Department of Biostatistics Vanderbilt University Medical Center Nashville Tennessee USA

**Keywords:** area under receiver operating characteristic curve, early detection, miRNA, pancreatic cancer, pre‐diagnostic plasma sample

## Abstract

Early diagnosis of pancreatic ductal adenocarcinoma (PDAC) is critical to improving the overall prognosis. However, biomarkers for early diagnosis are lacking. We used pre‐diagnostic plasma samples from 1307 PDAC cases and individually matched cancer‐free controls from five prospective cohort studies. Conditional logistic regression was used to assess the association of circulating miRNAs with PDAC risk. Linear regression was conducted to identify miRNAs ratio (miRNA levels of case over their matched control) change associated with the lead time from blood draw to PDAC diagnosis. We found 20 miRNAs significantly associated with PDAC risk (*p*_FDR_ < .05). Among them, 13 miRNAs showed a significant ratio change within 10 years prior to cancer diagnosis. The case–control difference of miR‐155‐5p and miR‐493‐3p increased when blood draw was closer to PDAC diagnosis. While the ratio changes of the remaining 11 miRNAs decreased. Adding 13 miRNAs to known risk factors and CA19‐9 significantly improved the prediction performance for imminent PDAC risk, with AUCs of 85%, 77%, and 73% during the time windows of 1, 2, and 3 years prior to PDAC diagnosis. Findings of this large prospective study shield a light on the biology of PDAC and suggest a potential utility of monitoring circulating miRNAs for PDAC risk surveillance among high‐risk individuals.

## Graphical abstract

What's new?

Early diagnosis is critical for improving pancreatic ductal adenocarcinoma prognosis, but few biomarkers currently exist. In a case–control study of 1,307 pancreatic cancer cases and matched pairs nested in prospective cohort studies, 13 miRNA–cancer associations were found to change during the blood‐draw‐to‐diagnosis time window. The area under the curves of these 13 miRNAs combined with CA19‐9 and pancreatic ductal adenocarcinoma risk factors were 85%, 77%, and 73% for the 1‐, 2‐, and 3‐year lead times, respectively. Circulating levels of miRNAs could potentially help identify individuals at imminent risk of developing pancreatic cancer through targeted screening and close surveillance.

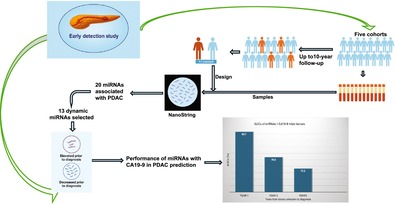

**Abbreviations**
AUC: area under receiver operating characteristic curve
MEC: the Multiethnic Cohort Study
PC: pancreatic cancer
PDAC: pancreatic ductal adenocarcinoma
PLCO: The Prostate, Lung, Colorectal, and Ovarian Cancer Screening Trial
SCCS: The Southern Community Cohort Study
SMHS: The Shanghai men's Health Study
SWHS: The Shanghai Women's Health Studies

## 1 INTRODUCTION

Pancreatic cancer (PC) is one of the most fatal cancers, with an overall five‐year survival rate near 13% in 2024.^1^ Among PCs, pancreatic ductal adenocarcinoma (PDAC) accounts for over 90% of cases.^2^ It is estimated that there were 67,440 new PC cases and 51,980 deaths in the US in 2025.^3^ Increasing trends in both incidence and mortality of PC were observed in most countries across the world.^4^ Early diagnosis of PC is critically important to improving the overall prognosis. However, biomarkers for early diagnosis are lacking.

MicroRNAs (miRNAs) are a class of small, single‐stranded and non‐coding RNAs that are involved in a series of developmental and physiological processes through regulating gene expression at the post‐transcriptional level.^5^, ^6^ Multiple miRNAs were found to be associated with various diseases, playing important roles in oncogenesis, cancer progression, metastasis, and drug resistance.^7^, ^8^ Recent studies suggested that miRNAs may play a role in the early detection of PC.^9^ However, many of these studies were limited by a small number of miRNAs evaluated and/or small sample sizes and often lacked racial/ethnic diversity among research participants. Importantly, few studies have prospectively investigated the relationship of PDAC risk with the dynamics of pre‐diagnostic miRNA levels, which may be important for PC early detection. In the current study, we used data from case–control studies nested in five prospective cohorts: the Prostate, Lung, Colorectal, and Ovarian Cancer Screening Trial (PLCO), the Shanghai Women's Health Study (SWHS) and Shanghai Men's Health Study (SMHS), the Southern Community Cohort Study (SCCS), and the Multiethnic Cohort Study (MEC) to address the research gap. We hypothesize that changes in miRNA levels during the period preceding PC diagnosis would be informative for PDAC early detection.

## 2 MATERIALS AND METHODS

### 2.1 Study population

**FIGURE 1:**
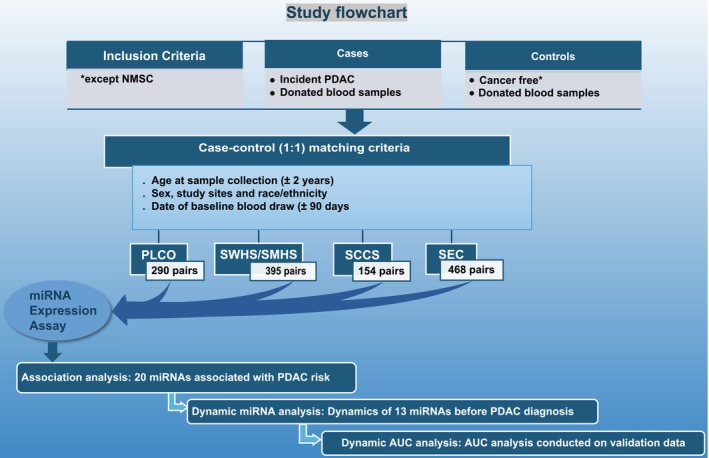
Flowchart. Study flowchart illustrating participant selection, matching criteria, and analysis steps in the nested case–control study.

Study participants were drawn from the PLCO, SCCS, SWHS, SMHS, and MEC, and included individuals of African, Asian, and European descent. Details of the parent cohort studies have been described elsewhere.^10^ Briefly, the PLCO was a large randomized controlled trial to determine whether certain screening exams reduce mortality from prostate, lung, colorectal, and ovarian cancer. About 155,000 participants, aged 55–74 years, were enrolled between November 1993 and July 2001 with 85.6% being White and 5%, Black. Vital status of participants and cancer occurrence were mainly identified via self‐reports in an annual mail‐in survey, state cancer registries and death certificates. Non‐fasting plasma samples were collected at baseline survey and stored at −70°C. The SCCS recruited about 85,000 participants aged 40–79 years between 2001 and 2009 in the southeastern United States, with over 80% being Africa American. Incident cancer cases and death status were determined through annual linkage with State Cancer Registries, the Social Security Administration, and the National Death Index (NDI). Non‐fasting plasma samples were collected from 51% participants at baseline survey and stored at −80°C. The SWHS and SMHS enrolled 74,940 women aged 40–70 years from 1997 to 2000 and 61,496 men aged 40–74 years from 2002 to 2006. Both women and men lived in urban Shanghai, China. Incident cancer cases and death status were determined by in‐person follow‐up surveys taking place every 2–4 years and annual record linkage to databases of the population‐based Shanghai Tumor Registry, the Shanghai Vital Statistics Registry, and the Shanghai Resident Registry. Non‐fasting plasma samples were collected for 75.8% in SWHS and 75.1% in SMHS at baseline survey respectively and stored at −70°C. The MEC consists of men and women primarily of five ethnic groups [Caucasians (22.9%), Japanese Americans (26.4%), Native Hawaiians (6.5%), African Americans (16.3%) and Latinos (22.0%)] with more than 215,000 participants. Incident cancer cases and survival status of participants were identified by cancer registries of the Surveillance, Epidemiology and End Results (SEER) Program through computer linkages and linkages to Medicare and NDI. From 2001 to 2006, fasting plasma samples of ~67,000 subjects were drawn using heparinized tubes, processed and separated; the 0.5 mL aliquots of plasma were stored in the vapor phase of liquid nitrogen (−150°C). In the current study we used 1307 pre‐diagnostic blood samples of primary pancreatic cancer cases (ICDO‐3: C25.0‐C25.3, C25.7‐C25.9) and their individually matched controls from the five cohorts. Controls were randomly selected from individuals free of any type of cancer, except non‐melanoma skin cancer (NMSC), and matched individually to each case (1:1 ratio) on age at sample collection (±2 years), sex, study site, race/ethnicity, and date of baseline blood draw (±90 days) within each cohort. A study flowchart is shown to clarify study design and data analysis in Figure 1. Among the 2614 participants included in the current study, 290 case–control pairs were from the PLCO, 395 pairs from the SWHS/SMHS, 154 pairs from the SCCS, and 468 pairs from the MEC. Median of storage duration of the study samples is 3.75 years (IQR: 1.76–6.63 years) for PLCO, 6.50 years (IQR: 4.00–10.00 years) for SCCS, 9.42 years (IQR: 5.89–12.32 years) for SWHS and SMHS, and 8.02 years (IQR: 4.32–11.28 years) for MEC. Additionally, a subset of 330 participants (428 case–control sample pairs) from the PLCO had miRNA measured in repeated blood samples collected at different time points (two‐repeated samples for 134 participants and three repeated samples for 196 participants). Information on BMI (derived from weight/height [kg/m^2^]), smoking status, history of diabetes, and family history of pancreatic cancer was collected at parent cohorts' baseline survey.

### 2.2 RNA extraction, miRNA profiling, data processing and analysis

#### 2.2.1 Total RNA extraction from plasma

Total RNA, including miRNA, was extracted and purified from plasma samples using the miRNeasy Serum/Plasma Kit (Qiagen, Valencia, CA), following the manufacturer's protocol. To minimize cellular contamination, plasma samples were centrifuged at 16,000 × *g* for 10 min at 4°C, and 200 μL of the supernatant was used for RNA extraction. To control for variability in starting material and extraction efficiency, 5 μL each of three synthetic spike‐in RNA oligonucleotides‐osa‐miR414 (2 pg/μL), cel‐miR248 (1 pg/μL), and ath‐miR159a (4 pg/μL)‐were added to each sample. From 20 μL of total RNA yield, 3 μL was used for miRNA expression analysis, representing RNA extracted from 30 μL of plasma.

#### 2.2.2 miRNA expression assays

miRNA expression was measured using the NanoString nCounter Human v3 miRNA Expression Assay (NanoString Technologies, Seattle, WA) which covers 798 common human miRNAs. This platform enables sensitive, reproducible, and multiplexed detection of specific miRNAs without the need for reverse transcription or amplification. The Human v3 assay includes six positive and eight negative assay controls, five non‐mammalian spike‐in probes (ath‐miR159a, cel‐miR248, cel‐miR254, osa‐miR414, and osa‐miR442). All assays were performed at the Vanderbilt Molecular Epidemiology Core Laboratory following NanoString's standard protocol. To minimize technical variation, matched case–control samples were processed in the same batch and assayed on the same cartridge.

#### 2.2.3 Quality control and data normalization

NanoString's assay Quality Control (QC, QC statistics for each sample are summarized in Table S1) guidelines were followed to ensure optimal ligation, hybridization, and imaging. Samples failing QC metrics (e.g., low field‐of‐view counts or abnormal binding density) were repeated.

miRNA data were normalized in three steps: (1) Background correction: non‐specific binding was removed by subtracting the mean signal from seven negative control probes. (2) Spike‐in normalization: counts were normalized using the mean expression of three synthetic spike‐in miRNAs (ath‐miR159a, cel‐miR248, and osa‐miR414) to account for variation in input material and extraction efficiency. (3) Sample content normalization: geometric mean counts of the top 50 expressed miRNAs were used, excluding hsa‐miR‐320e, hsa‐miR‐16‐5p, and hsa‐miR‐451a due to potential red blood cell contamination or hemolysis.^11^

### 2.3 Statistical analysis

The characteristics of cases and controls were compared by paired *t*‐test for continuous variables and conditional logistic regression for categorical variables within each study. Quantile normalization for miRNA expression data was applied to improve the distribution of expression values across samples. The associations between circulating miRNAs (per decile change) and PDAC (OR and 95% CI) were evaluated using conditional logistic regression, with adjustment for age at sample collection, BMI, smoking status, history of diabetes, and family history of pancreatic cancer. *p*‐Values were corrected for multiple testing using the False Discovery Rate (FDR) method. Ratios of miRNA were calculated using levels of miRNA from cases divided by their respective matched controls. A linear regression model was applied to evaluate the association between log (ratio) of miRNA and lead time which was defined as years from blood draw to PDAC diagnosis of cases. A linear mixed model was used to evaluate the ratios and the lead time association among PLCO participants with repeated miRNA measurements at different time points prior to PDAC diagnosis. Using known risk factors of PDAC, CA19‐9, and miRNAs with significant log (ratio), four different models were applied in time‐dependent areas under the receiver operating characteristic curve (AUC) calculation and comparison: Model 1 included age at sample collection and known PDAC risk factors, including BMI, smoking status, history of diabetes, and family history of pancreatic cancer. Model 2 included model 1 covariates and CA19‐9. Model 3 included model 1 covariates and the PDAC associated miRNAs, and model 4 included all predictors of model 1, model 2, and model 3. Internal validation with bootstrap method was applied to improve the robustness of assessments. All statistical analyses were performed using SAS office analysis (Version 7.12) and R (Version 4.5.1), and all statistical tests were based on a two‐sided significance level at *α* = 0.05.

## 3 RESULTS

Characteristics of participants from the five cohorts differed substantially (Table 1). Participants were predominantly White in the PLCO and Black in the SCCS. The SWHS and SMHS participants were exclusively Asians. In the MEC, 15% participants were White, 19% were Black, 37% were Japanese American, and 29% were Native Hawaiian or Latino. Mean age at diagnosis was highest in the MEC and lowest in the SCCS. The average number of years from blood draw to diagnosis for cases was highest in the SWHS/SMHS (median = 9.4 years) and lowest in the PLCO (median = 3.7 years). Characteristics between cases and controls were generally similar within each cohort, except those cases who had higher diabetes prevalence than controls in the PLCO, SWHS, SMHS, and SCCS. Also, cases in the PLCO had more current smokers than controls. The median lead time was 7.13 years (range: 0.03–19.61 years).

**TABLE 1:** Characteristics of pancreatic cancer cases and controls across studies.

	PLCO			SWHS/SMHS			SCCS			MEC		
	Cases	Controls	*p* ^a^	Cases	Controls	*p* ^a^	Cases	Controls	*p* ^a^	Cases	Controls	*p* ^a^
Number of subjects	290	290		395	395		154	154		468	468	
Age at sample collection, mean ± SD	68.0 ± 5.3	67.8 ± 5.3	.69	60.6 ± 9.0	60.8 ± 8.9	.77	56.3 ± 9.6	56.0 ± 9.8	.78	69.2 ± 8.0	69.2 ± 8.0	.98
Age at cancer diagnosis, mean ± SD	72.3 ± 6.3			69.8 ± 9.4			63.0 ± 9.8			77.6 ± 8.2		
Age at cancer diagnosis, *N* (%)												
<65 years	64 (11)			250 (32)			182 (59)			68 (7)		
≥65 years	516 (89)			540 (68)			126 (41)			868 (93)		
Years from sample collection to diagnosis, median (q1, q3)	3.7 (1.8, 6.6)			9.4 (5.9, 12)			6.5 (4.0, 10)			8.2 (4.3, 11)		
Years from sample collection to cancer diagnosis, *N* (%)												
<5 years	185 (64)			77 (19)			59 (38)			141 (30)		
5–10 years	86 (30)			142 (36)			55 (36)			165 (66)		
>10 years	19 (6)			176 (45)			40 (26)			162 (34)		
BMI, mean ± SD	27.1 ± 4.7	26.7 ± 3.9	.19	24.5 ± 3.3	24.2 ± 3.4	0.31	29.8 ± 7.8	29.7 ± 6.9	0.88	26.7 ± 4.6	26.5 ± 4.9	.52
BMI			.33			.82			.56			.51
Underweight	4 (1.4)	1 (0.3)		13 (3.3)	15 (3.8)		6 (3.9)	2 (1.3)		3 (0.7)	5 (1.1)	
Normal	94 (32.4)	107 (36.9)		214 (54.2)	225 (57.0)		36 (23.4)	37 (24.0)		177 (37.8)	191 (40.8)	
Overweight	129 (44.5)	129 (44.5)		146 (37.0)	135 (34.2)		53 (34.4)	51 (33.1)		201 (42.9)	180 (38.5)	
Obesity	63 (21.7)	53 (18.3)		22 (5.5)	20 (5.0)		59 (38.3)	64 (41.6)		87 (18.6)	92 (19.6)	
Sex, *N* (%)			1.00			1.00			1.00			1.00
Female	123 (42)	123 (42)		207 (52)	207 (52)		74 (48)	74 (48)		255 (54)	255 (54)	
Male	167 (58)	167 (58)		188 (48)	188 (48)		80 (52)	80 (52)		213 (46)	213 (46)	
Race/ethnicity, *N* (%)			1.00			1.00			.71			1.00
White	259 (89)	259 (89)		0 (0)	0 (0)		36 (23)	36 (23)		70 (15)	70 (15)	
Black	9 (3)	9 (3)		0 (0)	0 (0)		114 (74)	114 (74)		89 (19)	89 (19)	
Asian	0 (0)	0 (0)		395 (100)	395 (100)		0 (0)	0 (0)		174 (37)	174 (37)	
Others	22 (8)	22 (8)		0 (0)	0 (0)		4 (3)	2 (3)		135 (29)	135 (29)	
Smoking status, *N* (%)			<.001			.31			.09			.15
Never	115 (40)	153 (53)		262 (66)	282 (71)		42 (27)	53 (34)		235 (50)	244 (52)	
Former	120 (41)	117 (40)		29 (7)	24 (6)		34 (22)	42 (27)		175 (37)	184 (39)	
Current	55 (19)	20 (7)		104 (27)	89 (23)		78 (51)	59 (39)		58 (12)	40 (9)	
Diabetes at baseline, *N* (%)			.007			.007			.05			.23
No	257 (89)	275 (95)		351 (89)	372 (94)		105 (68)	120 (78)		452 (91)	435 (93)	
Yes	11 (11)	15 (5)		744 (11)	23 (6)		49 (32)	34 (22)		43 (9)	33 (7)	
Family history of pancreatic cancer, *N* (%)			1.00			1.00			.50			.11
No	283 (97)	283 (97)		387 (98)	389 (98)		152 (99)	154 (100)		454 (97)	462 (99)	
Yes	7 (3)	7 (3)		8 (2)	6 (2)		2 (1)	0 (0)		14 (3)	6 (1)	

Abbreviations: BMI, body mass index; MEC, multiethnic cohort; PLCO, Prostate, Lung, Colorectal and Ovarian Cancer Screening Trial; SWHS/SMHS, Shanghai Women's Health Study and Shanghai Men's Health Study; SCCS, Southern Community Cohort Study; SD, standard deviation.

^a^ *p*‐Values are calculated by paired *t*‐test for continuous variables and conditional logistic regression for categorical variables.

We conducted the association analysis stratified by lead time: <5 years (462 case–control pairs), 5–10 years (448 case–control pairs), and >10 years (397 case–control pairs). We found 86 miRNAs that were significantly associated with PDAC risk in the first‐time window. Among them, 20 had a *p* < .05 after FDR correction: let‐7b‐5p, let‐7 g‐5p, let‐7i‐5p, miR‐15b‐5p, miR‐18a‐5p, miR‐20a‐5p+miR‐20b‐5p, miR‐22‐3p, miR‐93‐5p, miR‐106a‐5p+miR‐17‐5p, miR‐106b‐5p, miR‐155‐5p, miR‐181a‐5p, miR‐191‐5p, miR‐199a‐5p+miR‐199b‐5p, miR‐223‐3p, miR‐340‐5p, miR‐451a, miR‐493‐3p, miR‐640, and miR‐1976 (Table 2). Only four miRNAs, miR‐155‐5p, miR‐493‐3p, miR‐640, and miR‐1976, were positively associated with PDAC risk, with ORs ranging from 1.11 to 1.14, while the rest were inversely associated, with ORs of 0.82–0.91. We did not find any significant ORs within the 5–10 years and >10 years windows after FDR correction, although let‐7 g‐5p, miR‐15b‐5p, and miR‐493‐3p reached nominal *p* < .05.

**TABLE 2:** Association of miRNAs and pancreatic cancer diagnosed within 5 years.

miRNA	Accession	OR (95% CI)^a^	*p*	*p*_FDR_
let‐7b‐5p	MIMAT0000063	0.87 (0.81–0.93)	1.26E−04	1.49E−02
let‐7 g‐5p	MIMAT0000414	0.90 (0.84–0.96)	1.04E−03	4.38E−02
let‐7i‐5p	MIMAT0000415	0.82 (0.74–0.90)	7.94E−05	1.27E−02
miR‐15b‐5p	MIMAT0000417	0.87 (0.80–0.94)	3.29E−04	2.61E−02
miR‐18a‐5p	MIMAT0000072	0.90 (0.84–0.96)	9.02E−04	4.23E−02
miR‐20a‐5p + miR‐20b‐5p	MIMAT0000075	0.83 (0.76–0.92)	1.31E−04	1.49E−02
miR‐22‐3p	MIMAT0000077	0.86 (0.78–0.94)	1.03E−03	4.38E−02
miR‐93‐5p	MIMAT0000093	0.85 (0.77–0.93)	3.94E‐04	2.61E‐02
miR‐106a‐5p + miR‐17‐5p	MIMAT0000103	0.84 (0.77–0.91)	3.44E−05	1.21E−02
miR‐106b‐5p	MIMAT0000680	0.91 (0.87–0.97)	1.15E−03	4.61E−02
miR‐155‐5p	MIMAT0000646	1.14 (1.06–1.22)	2.15E−04	2.15E−02
miR‐181a‐5p	MIMAT0000256	0.89 (0.84–0.95)	3.87E−04	2.61E−02
miR‐191‐5p	MIMAT0000440	0.82 (0.75–0.89)	3.20E−06	2.55E−03
miR‐199a‐3p + miR‐199b‐3p	MIMAT0000232	0.82 (0.75–0.91)	6.06E−05	1.21E−02
miR‐223‐3p	MIMAT0000280	0.86 (0.80–0.94)	3.58E−04	2.61E−02
miR‐340‐5p	MIMAT0004692	0.88 (0.82–0.95)	6.62E−04	3.53E−02
miR‐451a	MIMAT0001631	0.88 (0.82–0.95)	4.25E−04	2.61E−02
miR‐493‐3p	MIMAT0003161	1.12 (1.05–1.19)	6.63E−04	3.53E−02
miR‐640	MIMAT0003310	1.14 (1.07–1.21)	4.88E−05	1.21E−02
miR‐1976	MIMAT0009451	1.11 (1.04–1.18)	8.86E−04	4.23E−02

^a^ Adjusted for age at sample collection, BMI, medical history of diabetes, smoking status, and family history of pancreatic cancer.

**FIGURE 2:**
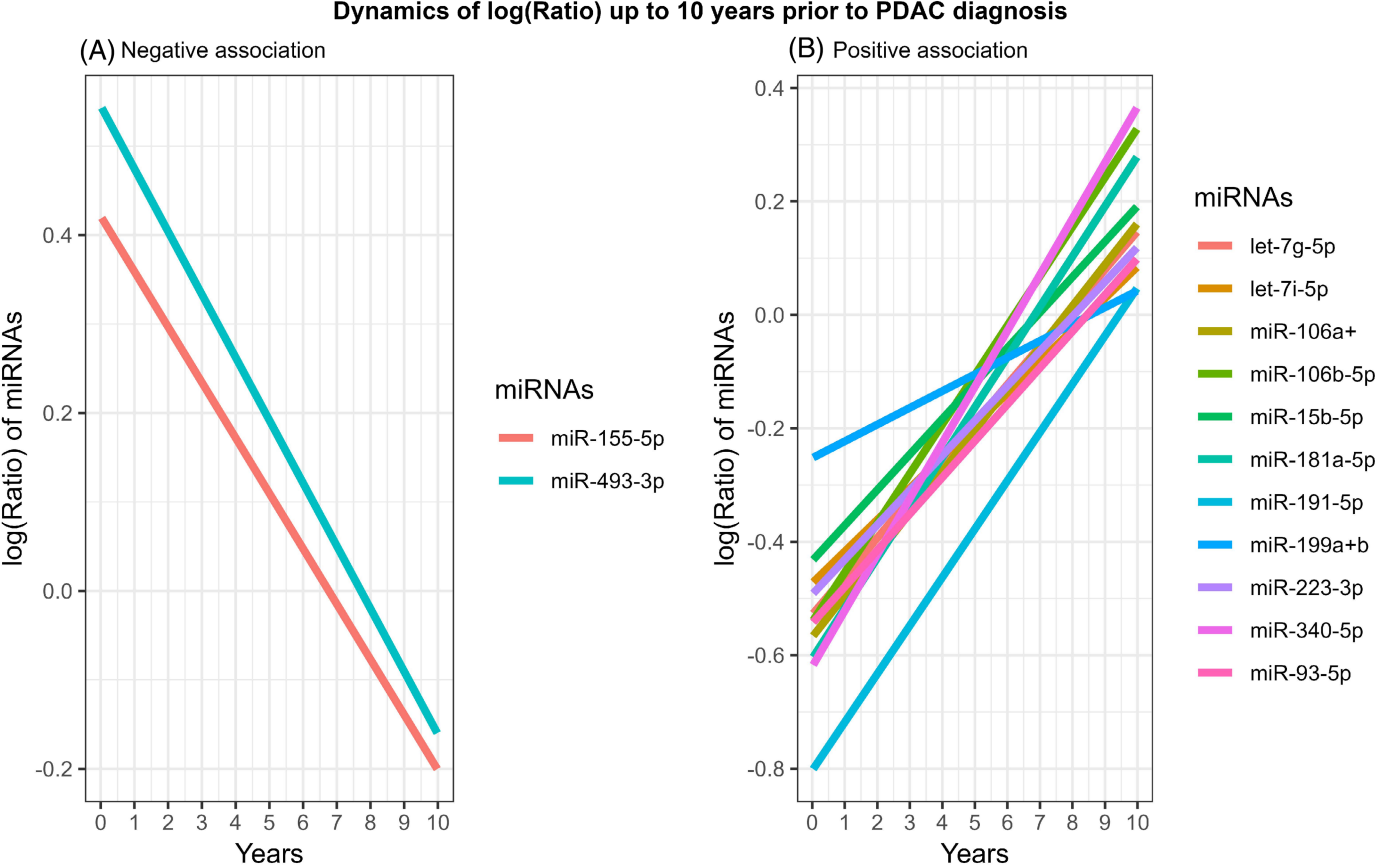
Dynamics of log(ratio) up to 10 years prior to PDAC diagnosis. (A) Negative association between log(ratio) of miRNAs and lead time (years). (B) Positive association between log(ratio) of miRNAs and lead time (years).

Table 3 shows that 13 out of the 20 FDR‐significant miRNAs had significant ratio changes in the 10‐year time window preceded PDAC diagnosis. We found that the ratios of miR‐155‐5p and miR‐493‐3p had a negative association with the lead time (*β* = −0.06 and *β* = −0.07, *p* trend <.05), that is, differences in miRNAs between cases and controls were larger when blood draw was closer to PDAC diagnosis. On the other hand, ratios of 11 miRNAs had a positive correlation with years from blood drawing to PDAC diagnosis, that is, the difference in miRNAs (cases/controls) decreased or even reversed (i.e., levels of miRNA are low in cases, compared to their matched controls) with the increase of the years from blood draw to PDAC diagnosis (Figure 2). Analysis using the data from subsets of PLCO participants with repeated miRNA measurements at multiple time points showed consistent direction for regression coefficients of all 13 miRNAs although only the association of four miRNAs, including let‐7i‐5p, miR‐181A‐5p, miR‐199a‐3p+miR‐199b‐3p and miR‐223‐3p, reached statistical significance, likely due to a much small sample size (Table S2).

**TABLE 3:** Association of ratios of miRNAs and up to 10 years of sample collection and PDAC diagnosis.

miRNA	Ratio of single measured miRNAs				
	Means^a^	SD^b^	Beta	SE	*p*
let‐7 g‐5p	−0.18967	2.58012	0.06671	0.02999	2.64E−02
let‐7i‐5p	−0.19328	1.43214	0.05073	0.01657	2.26E−03
miR‐15b‐5p	−0.12071	2.84524	0.07010	0.03313	3.46E−02
miR‐93‐5p	−0.2218	2.83169	0.06859	0.03282	3.69E−02
miR‐106a‐5p + miR‐17‐5p	−0.20257	2.79543	0.07356	0.03239	2.33E−02
miR‐106b‐5p	−0.10502	3.07616	0.08960	0.03578	1.24E−02
miR‐155‐5p	0.10918	2.00819	−0.06326	0.02333	6.80E−03
miR‐181a‐5p	−0.16192	3.16183	0.09358	0.0367	1.09E−02
miR‐191‐5p	−0.37669	2.81328	0.08622	0.03272	8.53E−03
miR‐199a‐3p + miR‐199b‐3p	−0.10454	1.26999	0.02939	0.01475	4.66E−02
miR‐223‐3p	−0.18569	2.23051	0.06275	0.02586	1.54E−02
miR‐340‐5p	−0.12536	2.65253	0.10080	0.03073	1.07E−03
miR‐493‐3p	0.19100	2.70659	−0.06726	0.03149	3.29E−02

^a^ Means of log(ratio).

^b^ SD of log(ratio).

**FIGURE 3:**
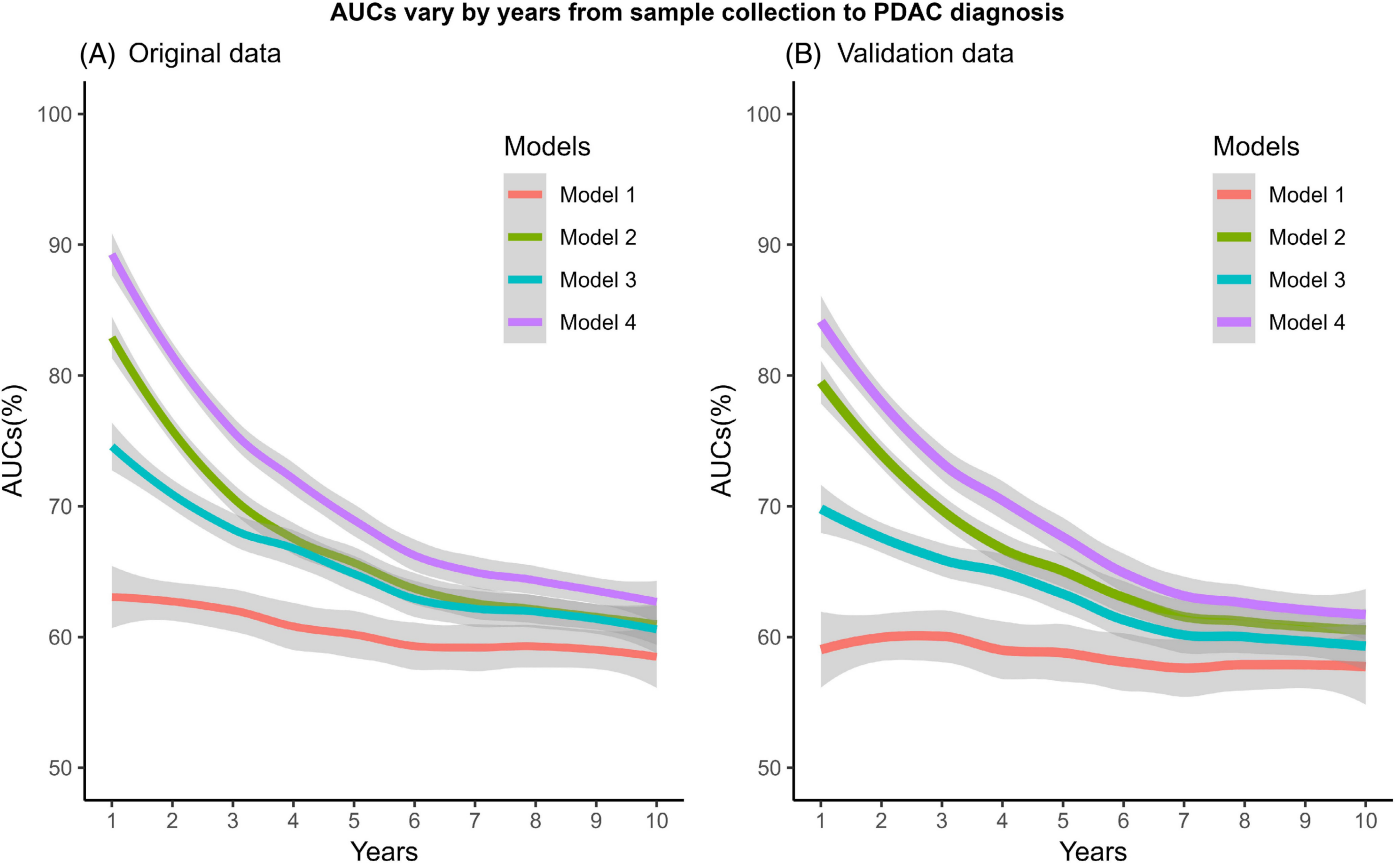
AUCs vary by years from sample collection to PDAC diagnosis. (A) AUCs calculated for each year using original data in each model. (B) AUCs calculated for each year using the average AUCs from bootstrapped testing data of each model. Below are predictors included in each model: Model 1: Age at sample collection, BMI, smoking status, history of diabetes, and family history of pancreatic cancer. Model 2: Model 1 + CA19‐9. Model 3: Model 1 + let‐7g‐5p + let‐7i‐5p + miR‐15b‐5p + miR‐93‐5p + miR‐106a‐5p/miR‐17‐5p + miR‐106b‐5p +. miR‐155‐5p + miR‐181a‐5p + miR‐191‐5p + miR‐199a‐3p/miR‐199b‐3p + miR‐223‐3p. miR‐340‐5p + miR‐493‐3p. Model 4: Model 1 + Model 2 + Model 3.

We derived four time‐dependent AUC curves based on known risk factors of PDAC, CA19‐9, and the 13 PDAC associated miRNAs with temporal changes (Figure 3B). The AUCs were 0.59, 0.62 and 0.59 at 1, 2, and 3 years preceding PDAC diagnosis based on model 1, which included only known PDAC risk factors. Adding CA19‐9 to model 1 increased prediction performance (years 1, 2, and 3 AUCs of 0.79, 0.75, and 0.69, respectively). Combination of the miRNAs and known risk factors (model 3) increased years 1, 2, and 3 AUCs to 0.70, 0.67, and 0.66. A combination of miRNAs, known risk factors and CA19‐9 (model 4) substantially improved the prediction performance, with years 1, 2, and 3 AUCs of 0.85, 0.77, and 0.73, respectively. The 95% confident interval (95% CI) of model 4 did not overlap with 95% CIs of other models within the lead time of the first 5 years, supporting model 4 being the best performance over the other models during this period. Furthermore, we estimated that CA19‐9 accounts for approximately 60.2% of the combined AUC in model 4 when the lead time is less than 1 year before PDAC diagnosis, providing quantitative context for its role in overall model performance during this interval. The 5‐year AUCs were 0.60 for model 1, 0.66 for model 2, 0.65 for model 3, and 0.69 for model 4.

We conducted a sensitivity analysis restricted to cases with early‐stage tumors (TNM: I/IIA) and their matched controls and observed higher AUCs (Table S3). We also conducted a sensitivity analysis only among cases who provided blood samples more than 1 year prior to PDAC diagnosis (Table S4). As expected, the AUCs in model 1, model 2, and model 4 decreased. However, the AUCs for model 3 (miRNA model) remained comparable to those observed in the analysis of all participants shown in Figure 3A.

## 4 DISCUSSION

In this large prospective case–control study, we found 20 circulating miRNAs that were significantly associated with PDAC diagnosed within 5 years after blood draw (*p*_FDR_ <.05). In the analysis of regressing the case/control ratio of these 13 risk associated miRNAs with the lead time between blood draw and PDAC diagnosis for cases during the 10 year window proceeding cancer diagnosis, two distinct patterns emerged (Figure 2): (1) the ratios of miR‐155‐5p and miR‐493‐3p were negatively associated with years of lead time, that is, the case–control difference was larger when blood draw was closer to PDAC diagnosis, suggesting their potential utility as early biomarkers for PDAC; and (2) the ratios of 11 miRNAs were positively correlated with years of lead time, that is, the ratios of 11 miRNAs decreased when assessment was taken close to cancer diagnosis, indicating they may be involved in early stages of carcinogenesis. No significant time trend was observed for the remaining miRNAs.

Cumulated evidence supports the idea that miRNAs play an important role in cancer development.^8^, ^12^ In a recent study, a panel of 165 cancer‐related miRNAs were identified based on various types of functional and genetic evidence.^13^ Ten out of thirteen miRNAs that showed a temporal change of association in our study are among the 165 cancer‐related miRNAs. Those miRNAs play a key role in the regulation of a wide range of cellular processes, including growth, differentiation, proliferation, and apoptosis.^14^ Also, they have been shown to have oncogenic or tumor‐suppressive function, and their overexpression or low expression were found in various cancers, including breast, lung, colorectal, pancreas, and liver cancers.^15^, ^16^

miR‐155‐5p is known as an oncogenic miRNA. It regulates genes that are located in sensitive sites of the genome, which are frequently amplified or deleted in the process of cancer development. This miRNA has been associated with pancreatic cancer^17^, ^18^, ^19^ and other cancers^20^ presumably through the dysfunctions of energy metabolism, avoiding elimination by immune cells, promoting inflammation, angiogenesis induction, and accumulation of mutations/instability of genetic material.^21^ We found that miR‐155‐5p was positively associated with risk of PDAC and its expression was stronger when the time of blood draw was close to the cancer diagnosis. This finding is supported by evidence from several other studies. One study reported that miR‐155 levels were significantly increased in intraepithelial neoplasia grade II pancreatic ductal epithelial cells, or early‐stage pancreatic cancer, in comparison to normal pancreatic tissues, suggesting that when malignant tumors occur, pancreatic ductal epithelial cells increase expression of miR‐155‐5p.^22^ Other studies have shown increased miR‐155‐5p expression in pancreatic cancer tissue, in comparison to normal pancreatic tissue or chronic pancreatitis tissue.^23^, ^24^, ^25^ These and our study findings support miR‐155‐5p's potential as a biomarker for early detection of PDAC.

Accumulating evidence shows that the expression level of miR‐493‐3p is associated with the occurrence of several cancers, and differential expression of miR‐493‐3p plays a vital role in cancer development, metastasis, and recurrence.^26^, ^27^ Functional studies from miR‐493 overexpression cells and nude mouse models revealed the tumor suppressor functions of miR‐493.^28^ The human either‐a‐go‐go‐related potassium channel 1 (hERG1) was identified as a direct target of miR‐493, which inhibits proliferation and invasion of PDAC and is generally reduced in pancreatic cancer tissue and cell lines.^14^, ^29^ Overexpression of miR‐493‐3p was reported to suppress ovarian cancer cell proliferation, migration, and invasion through downregulating DPY30.^30^ However, we found that high levels of miR‐493‐3p were positively associated with PDAC, and this association became stronger when the measurement was taken close to PDAC diagnosis. We did not find any report on expressions of miR‐493‐3p and PDAC risk. More studies are needed to understand the role of miR‐493‐3p in PDAC development and validate our findings.

For the 11 miRNAs whose expressions of PDAC cases became weaker when measurement took place close to cancer diagnosis, there is supportive biological evidence from both vitro and vivo models.^31^, ^32^, ^33^ Most of these miRNAs have cancer‐suppressor roles, although through different pathways for specific cancers: miR‐181a suppresses chronic leukemia partially via SERPINE1; miR‐223‐3p inhibits cell proliferation and migration via a miR‐223‐3p‐mutant p53 regulation feedback loop in lung cancer and miR‐199a can inhibit tumor growth and metastasis of lung cancer in vivo.^34^, ^35^, ^36^ However, miR‐106a‐5p has a bi‐directional role in cancer development; it is highly expressed in gastric, breast, and colorectal cancer, but is expressed at lower levels in squamous cell carcinomas, colon cancers, and gliomas. It was reported that miR‐106a promotes pancreatic tumorigenesis by directly targeting multiple genes involved in tumor suppression and cellular proliferation pathways.^37^ Whether miR‐106a‐5p is a tumor suppressive or oncogenic miRNA remains controversial, and the regulatory mechanism underlying miR‐106a‐5p‐mediated function, as well as the mechanism of its regulation, still requires elucidation in different cancers.^38^ Additionally, studies showed that miRNA levels of the let‐7 family were decreased in human PDAC^39^ and miR‐223‐3p was repressed in hepatocellular carcinoma and leukemia,^40^ but overexpressed in colorectal cancer and recurrent ovarian cancers.^41^ We found that miR‐223‐3p was negatively associated with PDAC risk, which is consistent with findings from Schultz's study and was one of the predictors included in their predictive models.^42^ We have previously reported that miR‐191‐5p and miR‐199a‐3p/199b‐3p were negatively associated with PDAC risk.^10^ They were reported as tumor suppressors and played crucial roles in suppressing cell proliferation and migration in various cancers, such as lung^43^ and PDAC.^44^ In the current study, approximately a decade before PDAC diagnosis, the case/control ratios for miR‐199a‐3p/199b‐3p and miR‐191‐5p were near unity. These ratios progressively declined to 0.78 and 0.46, respectively, reflecting a 25% reduction in miR‐199 and a 50% reduction in miR‐191‐5p as diagnosis approached, assuming stable miRNA levels in controls. In contrast, levels of the case/control ratio in miR‐155 and miR‐493 nearly doubled, rising from ~0.80 to ~1.60. This contrasting “scissors‐like” trajectory between tumor‐suppressive and oncogenic miRNAs highlights their potential utility as dynamic biomarkers for early PDAC detection.

An animal study reported dynamic changes in miRNA expression at 10, 30, 40, and 50 weeks during pancreatic cancer progression: miR‐216 and miR‐217 declined progressively, whereas miR‐21, miR‐34c, miR‐146b, miR‐205, and miR‐223 increased substantially.^45^ Consistent with these observations, we identified two distinct patterns of circulating miRNAs prior to PDAC diagnosis: two miRNAs exhibited progressive increases, while 11 miRNAs showed a marked decline as diagnosis approached. These findings indicated the potential of circulating miRNAs as minimally invasive biomarkers for early detection and longitudinal monitoring of pancreatic cancer, which could enable earlier intervention and improve patient outcomes.

In a case–control study that included 409 pancreatic cancer cases, researchers have identified two diagnostic panels of 4 (Index I) and 10 (Index II) miRNA expressions respectively with AUCs of 0.86 and 0.93 in classifying PDAC.^44^ Recently Baba et al. reported that they found miRNAs with AUCs of 0.972 (0.928–0.996) and 0.963 (95% CI, 0.932–0.988) in the training and test sets, respectively. Its miRNA‐based diagnosis was superior to CA19‐9 in terms of classifying PDAC cases and controls, including early stage (i.e., TNM of I‐IIA) cases.^46^ Another study conducted in Japan found that a combination of 100 selected miRNAs and CA19‐9 could accurately discriminate pancreatic cancer from healthy controls, with an AUC of 0.99.^47^ Treekitkarnmongkol et al. reported that three‐miRNA signature (let‐7i‐5p, miR‐130a‐3p and miR‐221‐3p) could be used to discriminate PDAC from healthy controls independently, with AUCs of 0.97, 0.96, and 0.97 for stage I, II, and III–IV, respectively.^48^ All these studies primarily used tissue or blood samples from PDAC cases after cancer diagnosis. It is unknown whether miRNAs before diagnosis can identify subjects at high risk of developing PDAC. In 2018, Frankin et al. reported that none of the candidate miRNAs, either individually or in combination, were significantly altered in pre‐diagnostic plasma samples in their miRNA‐PDAC study and underscoring the need for prospective, large‐scale studies.^49^ Several years later, two studies incorporated pre‐diagnostic plasma samples into PDAC research. Wang et al. used samples collected within 5 years prior to PDAC diagnosis and identified three circulating miRNAs associated with PDAC risk.^10^ Treekitkarnmongkol and colleagues further reported that the discriminative performance of circulating miRNAs improved as an early detection approach, with the AUC increasing from 0.702 to 0.729 to 0.757 at 12, 6, and 3 months prior to PDAC diagnosis, respectively.^48^ Both studies support the potential utility of circulating miRNAs for the early detection of PDAC. In the current study, we found that the addition of pre‐diagnostic levels of 13 miRNAs to known PDAC risk factors and CA19‐9 substantially improved prediction of future PDAC risk, with AUCs reaching 0.85, 0.77, and 0.73 during 1, 2, and 3 years prior to PDAC diagnosis. Of note, our study applied an individually matched case–control design, which may have led to an underestimation of the AUC. These results highlight the translational potential of including these miRNAs in identifying individuals at an elevated risk of developing PDAC.

To our knowledge, our study is the largest study to investigate circulating miRNAs as potential biomarkers for PDAC early detection and risk assessment. Our data came from five prospective studies and included White, Black, and Asian populations, which enhanced the validity and generalizability of our study findings. However, the sample size for each time window evaluated is still small and very few subjects had repeated miRNA measurements, resulting in insufficient statistical power in analyses with repeated biomarkers. Another limitation is that we could not adjust for chronic pancreatitis (CP) and pancreatic neuroendocrine tumors because these data were missing or unavailable in some cohorts. CP is a well‐established risk factor for pancreatic cancer and involves distinct inflammatory processes. Failure to account for CP may introduce confounding, potentially reducing the specificity of the identified miRNA biomarkers for PDAC detection. Notably, a recent study by Singh and colleagues identified a set of miRNAs capable of distinguishing CP from PDAC,^50^ six of which overlap with our 13‐miRNA panel, suggesting that this overlap may help minimize the impact of this limitation. More studies, particularly those with repeated time serial miRNA measurements, are warranted.

In conclusion, this large‐scale multiethnic study found that the dynamics of circulating miRNAs are associated with PDAC risk. If confirmed, they can be used to monitor PDAC risk among individuals at high risk of developing PDAC, such as those with late onset diabetes or a history of pancreatic cysts.

## AUTHOR CONTRIBUTIONS

**Hui Cai:** Methodology; data curation; visualization; formal analysis; writing – original draft; investigation; validation; writing – review and editing; project administration; conceptualization; software. **Veronica Wendy Setiawan:** Conceptualization; methodology; resources; validation; writing – review and editing; investigation. **Xingyi Guo:** Formal analysis; visualization; methodology; writing – original draft; writing – review and editing; investigation. **Jie Wu:** Data curation; investigation; resources; writing – review and editing. **Rachael Stolzenberg‐Solomon:** Conceptualization; methodology; writing – review and editing; investigation. **Yu‐Tang Gao:** Data curation; resources; investigation; writing – review and editing. **Jordan Berlin:** Investigation; conceptualization; writing – review and editing; methodology. **Fei Ye:** Methodology; investigation; writing – review and editing; formal analysis; data curation. **Qiuyin Cai:** Conceptualization; investigation; data curation; resources; writing – review and editing. **Wei Zheng:** Conceptualization; writing – review and editing; investigation; supervision; methodology. **Xiao‐Ou Shu:** Methodology; conceptualization; investigation; funding acquisition; writing – review and editing; formal analysis; supervision.

## FUNDING INFORMATION

This study is supported by a grant from the National Cancer Institute at the National Institutes of Health (CA227133).

## CONFLICT OF INTEREST STATEMENT

Dr. Berlin serves on ad boards for Regeneron, ALX oncology, EMD Serono, Ipsen, Taiho, BeOne and on DSMB of AstraZeneca. The other authors declare no conflicts of interest.

## ETHICS STATEMENT

The participants included in the study provided written informed consent and ethnic approvals for parent studies were obtained from the relevant institutional review boards.

## Supporting information


**TABLE S1:** Quality control (QC) matrix for miRNA expression assay in Nanostring.
**TABLE S2:** Association between ratios of repeatedly measured miRNAs and lead time (up to 10 years) among a subset of PLCO participants.
**TABLE S3:** Changes of AUCs in the years prior to PDAC diagnosis (TNM = I and IIA).
**TABLE S4:** Changes of AUCs in the years prior to PDAC diagnosis (excluding those cases with lead time (year) <1 year).

## Data Availability

The miRNA data has been deposited to the NCBI Gene Expression Omnibus (GEO: GSE314577). Additional datasets used and/or analyzed during the current study are available from the corresponding author on reasonable request pending permission from each of the Cohort studies and with approval of the data use agreement.
