# Supplementary material for: Dynamics of the association between circulating levels of miRNA and pancreatic cancer risk through the years prior to pancreatic cancer diagnosis
Source: Int J Cancer. 2026 Mar 17;159(2):526–37. doi: 10.1002/ijc.70452 (PMC13193466; doi:10.1002/ijc.70452)
Supplement: Supplementary file 1 — TABLE S1: Quality control (QC) matrix for miRNA expression assay in Nanostring. TABLE S2: Association between ratios of repeatedly measured miRNAs and lead time (up to 10 years) among a subset of PLCO participants. TABLE S3: Changes of AUCs in the years prior to PDAC diagnosis (TNM = I and IIA). TABLE S4: Changes of AUCs in the years prior to PDAC diagnosis (excluding those cases with lead time (year) <1 year). [file IJC-159-526-s001.pdf]

Dynamics of the association between circulating level of miRNA and pancreatic cancer risk through the years prior to pancreatic cancer diagnosis

Hui Cai, Veronica Wendy Setiawan, Xingyi Guo, Jie Wu, Rachael Stolzenberg-Solomon, Yu-Tang Gao, Jordan Berlin, Fei Ye, Qiuyin Cai, Wei Zheng, Xiao-Ou Shu

Table of Supplementary contents

Supplementary Tables	Contents
Supplementary Table 1	Quality control (QC) matrix for miRNA expression assay in Nanostring 0.5199
Supplementary Table 2	Association of ratios of repeated measured miRNAs and lead time (up to 10 years) among the subset of PLCO participants
Supplementary Table 3	Changes of AUCs in the years prior to PDAC diagnosis (TNM=I and IIA)
Supplementary Table 4	Changes of AUCs in the years prior to PDAC diagnosis (excluding those cases with lead time (year) <1year)

Supplementary Table 1. Quality control (QC) matrix for miRNA expression assay in Nanostring

QC	Imaging Quality[1]	Binding Density[2]	Positive Control Linearity[3]	Ligation QC[4]	Normalization Factors[5]
Threshold	Percent FOV registration <75%	0.1-2.25	$R^2 > 0.95$	< mean+3×sd	0.1-10

[1] Assessment of fields of view successfully counted versus attempted.
[2] Evaluation of barcode binding per square micron to confirm optimal hybridization efficiency.
[3] Calculation of Pearson correlation (R^2) for positive controls to verify assay linearity.
[4] Assessing the efficiency and specificity of the ligation step in miRNA assays by using positive ligation controls
[5] Normalization factors were derived from the geometric mean of the top 50 robustly expressed miRNAs, with global normalization applied to reduce technical variation.

Supplementary Table 2. Association between ratios of repeatedly measured miRNAs and lead time (up to 10 years) among a subset of PLCO participants

miRNA	Ratio change of repeatedly measured miRNAs		
	Beta	se	P
let-7g-5p	0.0311	0.0482	0.5199
let-7i-5p	0.0974	0.0254	0.0002
miR-15b-5p	0.1094	0.0561	0.0534
miR-93-5p	0.0401	0.0592	0.5000
miR-106a-5p+miR-17-5p	0.0295	0.0578	0.6112
miR-106b-5p	0.0739	0.0466	0.1160
miR-155-5p	-0.0252	0.0306	0.4120
miR-181a-5p	0.1334	0.0414	0.0016
miR-191-5p	0.1012	0.0609	0.0991
miR-199a-3p+miR-199b-3p	0.0583	0.0197	0.0037
miR-223-3p	0.1306	0.0425	0.0026
miR-340-5p	0.0328	0.0578	0.5709
miR-493-3p	-0.0930	0.0538	0.0862

Supplementary Table 3. Changes of AUCs in the years prior to PDAC diagnosis*

Models	1 year	2 years	3 years	4 years	5 years	6 years	7 years	8 years	9 years	10 years
Model 1	54.44	76.18	65.79	62.93	55.25	54.83	55.46	54.18	54.40	54.85
Model 2	92.31	85.80	75.73	75.55	67.52	63.77	63.03	62.87	62.99	60.58
Model 3	100.00	84.17	80.44	79.82	76.81	71.01	69.11	69.03	68.54	67.52
Model 4	100.00	89.64	87.02	84.20	80.40	73.83	72.23	72.01	71.23	69.55

*all cases with TNM in (I/IIA)

Supplementary Table 4. Changes of AUCs in the years prior to PDAC diagnosis*

Models	1 year	2 years	3 years	4 years	5 years	6 years	7 years	8 years	9 years	10 years
Model 1	-	68.42	61.97	61.61	60.61	59.70	58.72	58.75	59.37	58.09
Model 2	-	73.31	65.27	64.06	62.73	60.31	60.56	60.36	59.98	59.00
Model 3	-	72.77	69.20	66.83	64.68	62.32	61.49	61.73	61.57	60.03
Model 4	-	77.08	72.27	68.99	66.01	63.14	62.41	62.67	61.77	60.56

*excluded those cases with lead time (year) <1 year
